# Sacral Curvature in Addition to Sacral Ratio to Assess Sacral Development and the Association With the Type of Anorectal Malformations

**DOI:** 10.3389/fped.2021.732524

**Published:** 2021-10-01

**Authors:** Zhen Chen, Lingling Zheng, Minzhong Zhang, Jie Zhang, Ruixue Kong, Yunpei Chen, Zijian Liang, Marc A. Levitt, Chin-Hung Wei, Yong Wang

**Affiliations:** ^1^Department of Radiology, Guangzhou Women and Children's Medical Center, Guangzhou Medical University, Guangzhou, China; ^2^Clinical Data Center, Guangzhou Women and Children's Medical Center, Guangzhou Medical University, Guangzhou, China; ^3^Department of Pediatric Surgery, Xinhua Hospital Affiliated to Shanghai Jiaotong University School of Medicine, Shanghai, China; ^4^Department of Pediatric Surgery, Guangzhou Women and Children's Medical Center, Guangzhou Medical University, Guangzhou, China; ^5^Department of Nursing, Shandong Medical College, Ji'nan, China; ^6^Center of Reproductive Medicine, Guangzhou Women and Children's Medical Center, Guangzhou Medical University, Guangzhou, China; ^7^Division of Colorectal and Pelvic Reconstruction, Children's National Hospital, Washington, DC, United States; ^8^School of Medicine, The George Washington University, Washington, DC, United States; ^9^Division of Pediatric Surgery, Department of Surgery, Shuang Ho Hospital, New Taipei City, Taiwan; ^10^Department of Surgery, School of Medicine, College of Medicine, Taipei Medical University, Taipei, Taiwan

**Keywords:** anorectal malformations, sacral defect, sacral ratio, sacral curvature, sacral development

## Abstract

**Introduction:** Sacral ratio (SR) is currently the only measurement to quantitatively evaluate sacral development in patients with anorectal malformations (ARM). This study proposes sacral curvature (SC) as a new indicator to qualitatively assess the sacrum and hypothesizes that sacral development, both quantitatively and qualitatively, can be an indicator to predict the type of ARM. The study aims to investigate the difference of SR and SC between ARM types and the association with the type of ARM.

**Methods and Materials:** This study was retrospectively conducted between August 2008 and April 2019. Male patients with ARMs were enrolled and divided into three groups based on the types of ARM: (1) rectoperineal fistulae, (2) rectourethral-bulbar fistulae, and (3) rectourethral-prostatic or rectobladder-neck fistulae. SC was measured in the sagittal views of an MRI or a lateral radiograph of the sacrum.

**Results:** Included in the study were 316 male patients with ARMs. SRs were 0.73 ± 0.12, 0.65 ± 0.12, and 0.57 ± 0.12 in perineal, bulbar, and prostatic/bladderneck fistula, respectively (*p* < 0.01). The SCs in perineal fistulae and bulbar fistulae were significantly higher than that in prostatic/bladderneck fistulae (0.25 ± 0.04, 0.22 ± 0.14, and 0.14 ± 0.18, *p* < 0.01). When SR ≥ 0.779, there was an 89.9% of possibility that the child has a perineal fistula. When SR ≤ 0.490 and SC ≤ 0, the possibilities of the child having prostatic/bladderneck fistulae were 91.6 and 89.5%, respectively. SC < 0 was also noted in 27 (27.8%), 19 (10.5%), and no (0%) patients of prostatic/bladderneck, bulbar, and perineal fistulae (*p* < 0.01), respectively. Sacral defect was noted in 63% of patients with SC ≤ 0, compared to none with SC > 0 (*p* < 0.01).

**Conclusions:** The higher the rectal level is in an ARM, the lower are the objective measurements of the sacrum. SC ≤ 0 is associated with sacral defects and implies a high likelihood of prostatic/bladderneck fistulae.

## Introduction

Anorectal malformation (ARM) is a congenital anomaly and comprises a diverse spectrum of anomalies not only involving the anal canal and pelvis but also extending to other organs (e.g., heart, kidney, bones, and spine). The concept of sacral ratio (SR) in ARM was first proposed by Pena in 1995 (1). SR is an objective measurement of sacral development that has been widely used since then. Numerous researchers were devoted to connecting SR to predicting fecal incontinence (2–4) and the presence of tethered cord (5). Recent reports have shown that SR is associated with urinary tract anomalies and neurogenic bladder (3, 6).

SR is an index of quantitative sacral development to evaluate the length of sacrum. However, no attention has been paid to qualitative sacral development, which could be represented by the curvature of the sacrum. The normal morphology of the sacrum is anteriorly curved. The curvature develops in the early stage of the embryo (7–9). It is not unusual to see a straight or posteriorly curved sacrum in patients with ARM, which we believe demonstrates a deficiency in sacral development from a qualitative point of view. The clinical significance of this finding has not been previously studied.

In the present study, sacral development was assessed not only by SR but also by sacral curvature (SC). We hypothesize that sacral development is different between ARM types and can be an indicator to predict of the types of ARM. Thus, this study aims to investigate the difference of SR and SC between ARM types and the association with ARM types.

## Methods

### Patient Selection and Grouping

This retrospective study was performed by reviewing the medical records and imaging studies of male patients diagnosed with ARM between August 2008 and April 2019 in Guangzhou Women and Children Medical Center, China. Male patients with ARM who had pelvic MRI or a lateral radiograph of the sacrum were enrolled. Conversely, male patients with ARM who had no MRI or a lateral radiograph of the sacrum available were excluded. The data collected comprised ages, ARM types, MRI, and x-rays. The types of ARM were defined by intraoperative findings according to the Krickenbeck classification (10). The patients were divided into three groups based on the type of ARM, (1) rectoperineal fistulae (perineal fistulae), (2) rectourethral bulbar fistula (bulbar fistulae), and (3) rectourethral prostatic or rectobladder neck fistulae (prostatic/bladderneck fistulae).

### Imaging and Measurement

**Figure 1:**
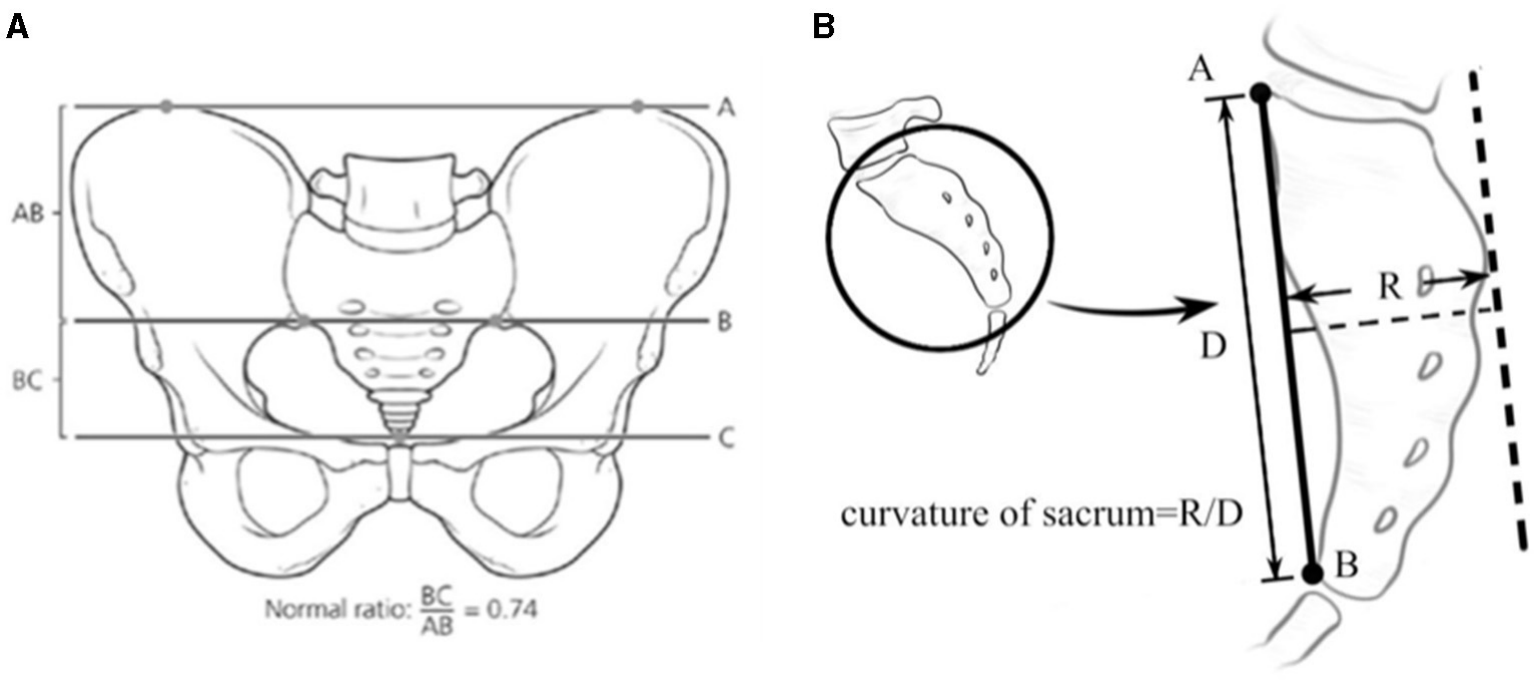
**(A)** The calculation of SR (6). **(B)** the measurement of SC.

SR was calculated in the anterior–posterior view of the pelvis x-rays with the classic formula proposed by Torres et al. (11) (Figure 1A).

**Figure 2:**
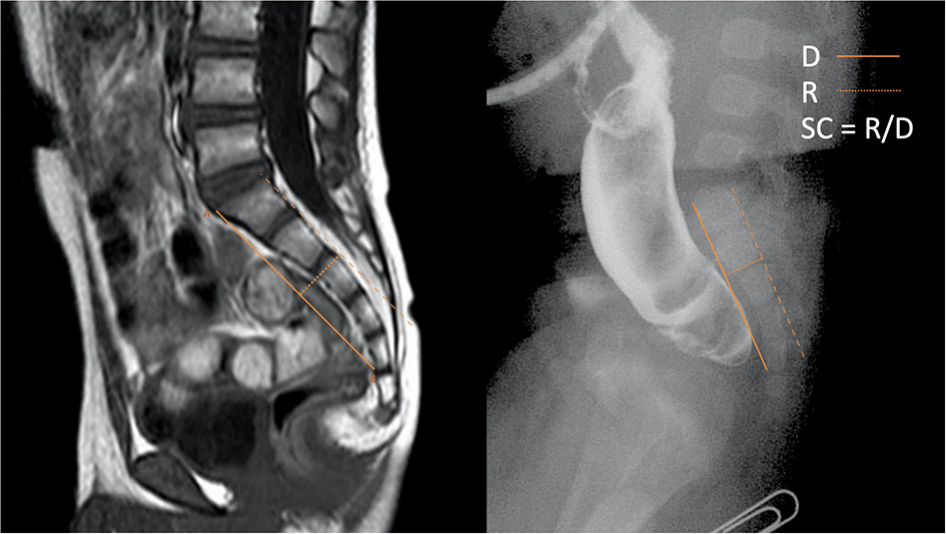
SC measurement in MRI and x-ray.

The measurement of SC is illustrated in Figure 1B. A-point is the junction of the superior and the ventral margin of the first sacral vertebra on the lateral viewed image; B-point is the junction of the inferior and the ventral margin of the fifth, or the last sacral vertebra. D indicates the distance between A and B. R represents the vertical distance from the highest point on the dorsal curve of the sacrum bending to the AB line. SC is the ratio of R to D. SC is measured in the sagittal views of the MRI or the lateral radiograph of the sacrum (Figure 2). When the sacrum is dorsally curved, the AB line is drawn on the dorsal side of the sacrum. SC is presented as a negative value. A dorsally curved sacrum was defined as SC ≤ 0.

The x-rays and MRI were retrospectively interpreted by the same pediatric radiologist team blinded to the clinical findings. The measurements of SR, *D, R*, and SC were also performed by the same team.

### Statistical Analysis

Descriptive statistics were used to evaluate the baseline demographic variables and the clinical characteristics of the image studies for all patients. Categorical and continuous variables were expressed as percentages and mean (standard deviation), respectively. All statistical analyses were performed using the MedCalc software (version 19.5.3). The three groups of ARMs were then compared in terms of the baseline demographic variables and the clinical characteristics of images using Fisher's exact test or χ^2^ test for categorical data and analysis of variance for continuous variables. Propensity scores were used to compare between groups. The correlation between age, SR, SC, *D*, and *R* was determined by Pearson's correlation. Moreover, the receiver operating characteristic (ROC) curves were constructed to distinguish between perineal fistulae and the other two groups as well as between bulbar fistulae and prostatic/bladderneck fistulae. Consequently, the areas under the curves (AUC) of the SR and SC were analyzed in a pairwise approach using the DeLong method. A *p* < 0.05 was considered to be statistically significant.

## Results

### Overall Characteristics and Group Comparison

Table 1 summarizes the patients' characteristics and comparison between groups. Moreover, this study analyzed the data of 316 male patients with ARMs. The overall mean age was 5.27 ± 5.22 months. Consequently, 38 (12.0%), 181 (57.2%), 54 (17.1%), and 43 (13.6%) patients had perineal, bulbar, prostatic, and bladderneck fistulae, respectively. The mean values of SR, *D, R*, and SC in the overall study population were 0.63, 5.15, 1.07, and 0.19 cm, respectively. Furthermore, SC was assessed by MRI in 24 (63.2%), 35 (19.3%), and 23 (23.7%) patients with perineal, bulbar, and prostatic/bladderneck fistulae, respectively (*p* < 0.01). The mean SCs calculated by MRI and x-rays were not significantly different in the overall population and groups (*p* > 0.05).

**Table 1:** Demographic and clinical characteristics.

	**Overall** ***n* = 316**	**Perineal** ***n* = 38**	**Bulbar** ***n* = 181**	**Prostatic/bladderneck** ***n* = 97**	***p^***a***^***	***p^***b***^***
Age (month)	5.27 ± 5.22	7.93 ± 8.47	4.76 ± 4.76	5.20 ± 4.00	<0.01	0.44
MRI (%)	25.9%	63.2%	19.3%	23.7%	<0.01	0.051
SR	0.63 ± 0.13	0.73 ± 0.12	0.65 ± 0.12	0.57 ± 0.12	<0.01	<0.01
D (cm)	5.15 ± 1.43	6.07 ± 1.17	4.83 ± 1.15	5.38 ± 1.76	<0.01	<0.01
R (cm)	1.07 ± 0.81	1.50 ± 0.40	1.07 ± 0.73	0.92 ± 1.00	<0.01	0.16
SC	0.20 ± 0.15	0.25 ± 0.04	0.22 ± 0.14	0.14 ± 0.18	0.18	<0.01

*p^a^ = Perineal vs. Bulbar.*

*p^b^ = Bulbar vs. Prostatic/bladderneck*.

The mean age in perineal fistulae was significantly higher compared with bulbar fistulae (7.93 ± 8.47 vs. 4.76 ± 4.76 months; *p* < 0.01). Furthermore, SR, *D*, and *R* also achieved statistical significance between the two groups. However, no difference in SC was noted. Consequently, age matching analysis was made as shown in Table 2. SR and SC were similar between both groups, and *D* and *R* in perineal fistulae remained significantly longer.

**Table 2:** Age-matching comparison between perineal and bulbar fistulae.

	**Perineal** ***n* = 18**	**Bulbar** ***n* = 18**	***p***
Age (month)	7.11 ± 3.36	7.06 ± 3.20	0.96
SR	0.73 ± 0.13	0.68 ± 0.15	0.341
D (cm)	6.28 ± 0.53	4.45 ± 0.91	<0.01
R (cm)	1.49 ± 0.30	1.04 ± 0.50	<0.01
SC	0.24 ± 0.05	0.22 ± 0.09	0.60

**Figure 3:**
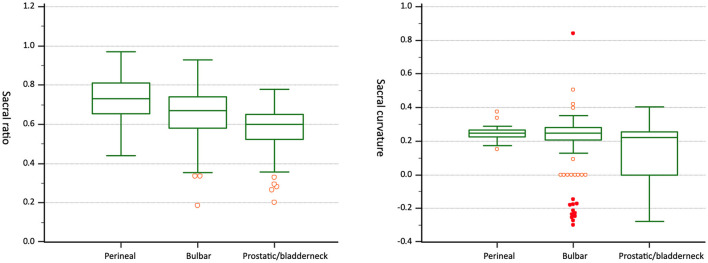
SR was significantly different between the three groups. SC of prostatic/bladderneck fistulae was significantly lower than those of perineal and bulbar fistulae.

The mean ages in bulbar fistulae and prostatic/bladderneck fistulae were 4.76 ± 4.76 and 5.20 ± 4.00 months (*p* = 0.44). SR, *D*, and SC achieved statistical significance between the two groups. However, no difference in *R* was noted. Figure 3 depicts SR and SC in the three groups.

### Correlation of Age, SR, SC, D, and R

The correlation of the variables is shown in Table 3. SR and SC were not associated with age in the overall population and all groups. However, *D* and *R* demonstrated a weak positive relationship to age in the overall population but varied between the groups. Similarly, the relationship between SR and SC was weak and also varied between the groups. A similar phenomenon was shown in the relationship of SR to *D* and *R*. Thus, SC had a universally strong association with *R* in the overall and all groups, but only with *D* in prostatic/bladderneck fistulae. Furthermore, a strongly positive correlation was noted between *D* and *R*.

**Table 3:** Correlation of age, SR, SC, D, and R.

	**Age**	**SR**	**SC**	**D**	**R**	
Age	Overall		0.023	0.091	0.232^**^	0.164^**^
	Perineal		−0.069	0.020	0.348^*^	0.285
	Bulbar		−0.068	0.046	0.180^*^	0.099
	Prostatic/bladderneck		0.132	0.189	0.174	0.200^*^
SR	Overall	0.023		0.354^**^	0.208^**^	0.370^**^
	Perineal	−0.069		0.182	0.222	0.268
	Bulbar	−0.068		0.314^**^	0.147^*^	0.354^**^
	Prostatic/bladderneck	0.132		0.295^**^	0.317^**^	0.321^**^
SC	Overall	0.091	0.354^**^		0.266^**^	0.909^**^
	Perineal	0.020	0.182		0.046	0.642^**^
	Bulbar	0.046	0.314^**^		0.131	0.909^**^
	Prostatic/bladderneck	0.189	0.295^**^		0.485^**^	0.941^**^
D	Overall	0.232^**^	0.208^**^	0.266^**^		0.593^**^
	Perineal	0.348^*^	0.222	0.046		0.792^**^
	Bulbar	0.180^*^	0.147^*^	0.131		0.460^**^
	Prostatic/bladderneck	0.174	0.317^**^	0.485^**^		0.717^**^
R	Overall	0.164^**^	0.370^**^	0.909^**^	0.593^**^	
	Perineal	0.285	0.268	0.642^**^	0.792^**^	
	Bulbar	0.099	0.354^**^	0.909^**^	0.460^**^	
	Prostatic/bladderneck	0.200^*^	0.321^**^	0.941^**^	0.717^**^	

* *p < 0.05;*

** *p < 0.01.*

*p > 0.7, strong correlation; 0.4–0.7, moderate correlation; p < 0.4, weak or correlation*.

### ROC Analysis

**Figure 4:**
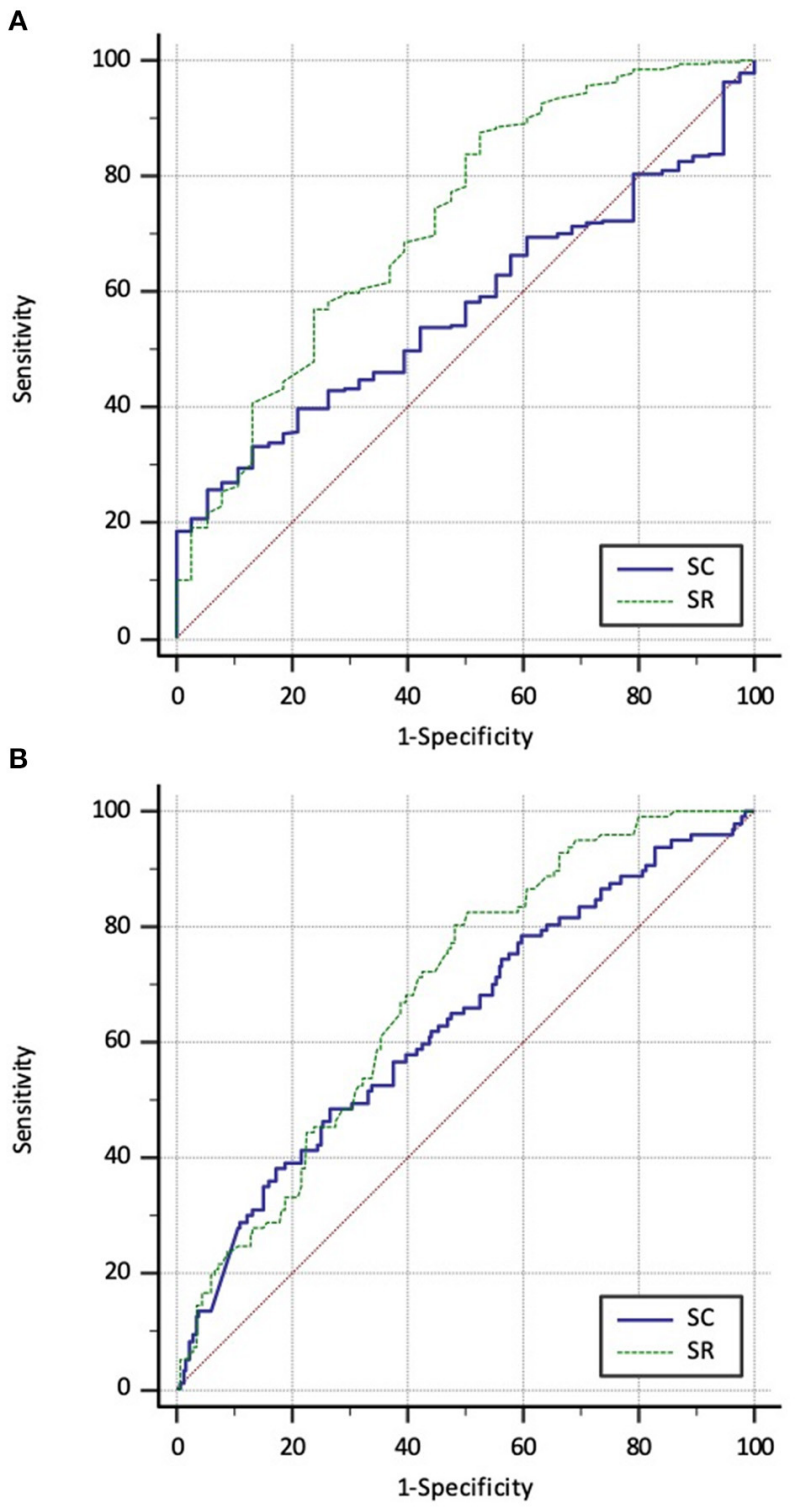
**(A)** Comparison of ROC curves of SR and SC for differentiating perineal fistulae from bulbar and prostatic/bladderneck fistulae (*p* < 0.01). **(B)** Comparison of ROC curves of SR and SC for differentiating bulbar fistulae from prostatic/bladderneck fistulae (*p* = 0.21).

Table 4 depicts the ROC curve for predicting bulbar and prostatic/bladderneck fistulae from perineal fistulae. Moreover, SR had a larger AUC compared with SC (0.72 vs. 0.57, *p* < 0.01; Figure 4A). Consequently, it demonstrated 87.4 and 47.4% of sensitivity and specificity, respectively, with 0.75 of SR. Furthermore, it showed 25.5 and 94.7% of sensitivity and specificity, respectively, with 0.19 of SC.

**Table 4:** ROC analysis for differentiation of bulbar fistulae and prostatic/bladderneck fistulae from perineal fistulae.

	**Cutoff point**	**Sensitivity**	**Specificity**	**+LR**	**–LR**	**AUC (95%CI)^a^**
SR	≤0.75	87.4%	47.4%	1.66	0.27	0.72 (0.67–0.77)^*^
SC	≤0.19	25.5%	94.7%	4.85	0.79	0.57 (0.51–0.63)
	**SR**	**Sensitivity %**	**Specificity %**	**SC**	**Sensitivity %**	**Specificity %**
	≤0.558	25.2	92.1	≤0.195	27.0	92.1
	≤0.560	26.2	89.5	≤0.197	27.0	89.5
	≤0.779	89.9	39.5	≤0.290	83.8	5.26
	≤0.780	91.0	36.8	≤0.336	96.4	5.26

*+LR = positive likelihood ratio; –LR = negative likelihood ratio.*

a *Compare to AUC = 0.5.*

* *p < 0.05*.

Table 5 depicts the ROC curve for predicting prostatic/bladderneck fistulae from bulbar fistulae. SR and SC had a similar AUC (0.69 vs. 0.63, *p* = 0.21; Figure 4B). It demonstrated 87.4 and 47.4% of sensitivity and specificity, respectively, with 0.75 of SR. Moreover, it showed 25.5 and 94.7% of sensitivity and specificity, respectively, with 0.19 of SC.

**Table 5:** ROC analysis for differentiation of prostatic/bladderneck fistulae from bulbar fistulae.

	**Cutoff point**	**Sensitivity**	**Specificity**	**+LR**	**–LR**	**AUC (95%CI)^a^**
SR	≤0.66	80.4%	51.9%	1.67	0.38	0.69 (0.63–0.74)^*^
SC	≤0.21	48.5%	73.5%	1.83	0.70	0.63 (0.57–0.69) ^*^
	**SR**	**Sensitivity %**	**Specificity %**	**SC**	**Sensitivity %**	**Specificity %**
	≤0.490	23.7	91.6	≤-0.146	13.4	93.9
	≤0.535	26.8	86.7	≤0.000	27.8	89.5
	≤0.700	88.7	35.9	≤0.289	89.7	19.3
	≤0.718	92.8	32.0	≤0.291	90.7	18.8

*+LR = positive likelihood ratio; –LR = negative likelihood ratio.*

a *Compare to AUC = 0.5.*

* *p < 0.05*.

### Dorsally Curved Sacrum

Forty-six patients had a dorsally curved sacrum, 27 (27.8%), 19 (10.5%), and no (0%) patients with prostatic/bladderneck, bulbar, and perineal fistulae, respectively (*p* < 0.01). All the patients with a dorsally curved sacrum were associated with an absent or hypoplastic coccyx. Sacral defects were found in 29 patients with a dorsally curved sacrum (63.0%), including partial or complete absence of vertebrae, hypoplastic vertebrae, vertebral fusion, and unfused laminae, but in none with a ventrally curved sacrum (*p* < 0.01). With 0 of SC, the analysis to differentiate bulbar fistulae from prostatic/bladderneck fistulae showed 27.8 and 89.5% of sensitivity and specificity, respectively.

## Discussion

Based on Krickenbeck classification, the types of male ARM are diagnosed by the location of the fistula instead of the high, intermediate, and low types. This study grouped the patients based on the classification. However, prostatic and bladderneck fistulae were combined into one group because the current consensus advocates that the laparoscopic approach is indicated for these two types (12, 13).

In the cohort of this study, the case number in perineal fistula was less than those in bulbar and prostatic/bladderneck fistulae. This finding was not compatible with the normal rate of the types of ARM. The institution where the study was conducted was a tertiary referral pediatric medical center. The majority of patients who were transferred to this institution had complex situations. Patients with perineal fistulae might tend to receive surgery in local institutions. Meanwhile, a large number of patients with perineal fistulae in the institution did not have an MRI or a lateral film of the sacrum. These factors contributed to these observations.

This is the first report to propose the concept of SC in ARM. SC was able to be calculated in this study by either MRI or x-rays by a specialized pediatric radiology team, and is a reproducible calculation. However, a variety of assessment modalities may affect the result. The analysis showed that the SCs of MRI and x-rays are similar. Practically, MRI and radiographs both serve as appropriate modalities for SC evaluation. Some pros and cons exist for each one. MRI provides better anatomical details beyond sacrum morphology, while radiographs are easily taken for multiple purposes (e.g., cross-table lateral film or distal colostogram) without the need for sedation.

The literature suggested that the normal value of SR is 0.74, which is significantly higher than the ARM patients (1, 11, 14). Nevertheless, the difference of the SRs in the ARM types is not clear. The SR in RPF in our cohort is 0.73, which is similar to the SR in normal children (11). It is also noted that SR is significantly different between the groups. The more complex the ARM type is, the lower the SR is. In terms of SC, the different is only noted between bulbar and prostatic/bladderneck fistulae. It was noticed that SR has acceptable discrimination between RPF and the other two groups (Table 4). With a probability of 90%, SR ≥ 0.779 suggested perineal fistulae and SR ≤ 0.560 suggested the other types. For the differentiation between bulbar and prostatic/bladderneck fistulae, both SR and SC only have low discrimination (Table 5). With a probability of 90%, SR ≥ 0.7 and SC ≥ 0.289 suggested bulbar fistulae, while SR ≤ 0.490 and SC ≤ 0 suggested prostatic/bladderneck fistulae.

A longitudinal observation demonstrated that SR declined when age increased although no statistics were made in this observation (11). Furthermore, Warne et al. proclaimed that the incomplete ossification of the sacrum in young infants may interfere with the interpretation of SR. This study showed that *D* and *R* increased with age overall, and SC and SR were not associated with age. The discrepancy between the current study and the literature might be due to the fact that the majority of the patients in this study were under the age of 1 year. The trend was not significant in this small range of the age. However, SC was not influenced by sacral ossification status because it did not measure coccyx as SR did.

In sacrum embryology, the ventral curve is the primary curvature of the embryo that develops in the early stage (9, 15, 16). The sacrum retains the curvature as the vertebral bodies grow up and increase (9). The size of the sacrum continues to shrink when the malformation become severe. However, the curvature was significantly lost in prostatic/bladderneck fistulae. The presence of a dorsally curved sacrum may imply that the anomaly starts in the early embryonal stage. Dorsally curved sacrum is highly associated with prostatic/bladderneck fistulae, and the finding supports that severe ARMs result from early developmental arrest in normal embryos (7, 8).

In addition to the association with prostatic/bladderneck fistulae, we also noted that no patient with perineal fistulae had a dorsally curved sacrum in this study. The recognition of a dorsally curved sacrum may serve as a reference for decision-making toward either colostomy or primary anoplasty, as well as posterior sagittal approach or laparoscopy. It should be emphasized that clinical inspection and distal colostogram remain gold standard for diagnosis. Meanwhile, the presence of dorsally curved sacrum also means a more than 60% chance of having a sacral defect.

This study focused on male rather than female ARMs. The diagnosis of male ARMs requires image studies or cystoscopy. However, in female ARM/cloaca patients, careful physical examinations can provide sufficient insights into the type of anomalies. Thus, sacral development will not play any role in the initial differentiation. Furthermore, some reports suggested that the sacrum does not develop equivalently in terms of straight length and the joint angle between the males and females (17, 18). Because of the anatomical differences, it is not clear if the findings in male ARM patients can be similarly revealed in female ARM patients. There has been no evidence showing the similarity of SR between male and female ARM patients. Consequently, more scientific pieces of evidence are needed to support it.

This study has several limitations as other retrospective ones do. The case number of the perineal fistulae is much less than the other two groups, which is not compatible with the general occurrence rate. The SC calculation was heterogeneously conducted by MRI or x-rays. A control group of normal populations for SC was absent. Thus, reproducibility and interrater reliability require further investigation. Moreover, this study did not investigate the association between SC and functional outcomes, which requires further work to study.

In conclusion, the results of this study show that SR and SC differ between the types of male ARMs. SC is a brand-new indicator to evaluate sacral development. A dorsally curved sacrum is associated with a low likelihood of perineal fistulae and a high likelihood of prostatic/bladderneck fistulae.

## Data Availability Statement

The raw data supporting the conclusions of this article will be made available by the authors, without undue reservation.

## Author Contributions

YW, LZ, and C-HW: study conception and design. YW, ZC, LZ, RK, and ZL: data acquisition. YW, ZC, YC, and C-HW: analysis and data interpretation. YW, LZ, JZ, and C-HW: drafting of the manuscript. ML, C-HW, and MZ: critical revision. All authors contributed to the article and approved the submitted version.

## Funding

This work was supported by grants from National Natural Science Foundation of China (Grant No. 81800448) and National Natural Science Foundation of China (Grant No. 81800110).

## Conflict of Interest

The authors declare that the research was conducted in the absence of any commercial or financial relationships that could be construed as a potential conflict of interest.

## Publisher's Note

All claims expressed in this article are solely those of the authors and do not necessarily represent those of their affiliated organizations, or those of the publisher, the editors and the reviewers. Any product that may be evaluated in this article, or claim that may be made by its manufacturer, is not guaranteed or endorsed by the publisher.
