# Corrigendum: Sacral Curvature in Addition to Sacral Ratio to Assess Sacral Development and the Association With the Type of Anorectal Malformations

**DOI:** 10.3389/fped.2022.922448

**Published:** 2022-05-06

**Authors:** Zhen Chen, Lingling Zheng, Minzhong Zhang, Jie Zhang, Ruixue Kong, Yunpei Chen, Zijian Liang, Marc A. Levitt, Chin-Hung Wei, Yong Wang

**Affiliations:** ^1^Department of Radiology, Guangzhou Women and Children's Medical Center, Guangzhou Medical University, Guangzhou, China; ^2^Clinical Data Center, Guangzhou Women and Children's Medical Center, Guangzhou Medical University, Guangzhou, China; ^3^Department of Pediatric Surgery, Xinhua Hospital Affiliated to Shanghai Jiaotong University School of Medicine, Shanghai, China; ^4^Department of Pediatric Surgery, Guangzhou Women and Children's Medical Center, Guangzhou Medical University, Guangzhou, China; ^5^Department of Nursing, Shandong Medical College, Ji'nan, China; ^6^Center of Reproductive Medicine, Guangzhou Women and Children's Medical Center, Guangzhou Medical University, Guangzhou, China; ^7^Division of Colorectal and Pelvic Reconstruction, Children's National Hospital, Washington, DC, United States; ^8^School of Medicine, The George Washington University, Washington, DC, United States; ^9^Division of Pediatric Surgery, Department of Surgery, Shuang Ho Hospital, New Taipei City, Taiwan; ^10^Department of Surgery, School of Medicine, College of Medicine, Taipei Medical University, Taipei, Taiwan

**Keywords:** anorectal malformations, sacral defect, sacral ratio, sacral curvature, sacral development

In the published article, there was an error in affiliation “10”. Instead of “Department of Surgery, School of Medicine, Taipei Medical University, Taipei, Taiwan”, it should be “Department of Surgery, School of Medicine, College of Medicine, Taipei Medical University, Taipei, Taiwan”

The authors apologize for this error and state that this does not change the scientific conclusions of the article in any way. The original article has been updated.

## Publisher's Note

All claims expressed in this article are solely those of the authors and do not necessarily represent those of their affiliated organizations, or those of the publisher, the editors and the reviewers. Any product that may be evaluated in this article, or claim that may be made by its manufacturer, is not guaranteed or endorsed by the publisher.

